# The Prevalence of High-Risk Prescribing of Oral Non-Steroidal Anti-Inflammatory Drugs in Primary Healthcare: A Single-Centre Retrospective Chart Review Study

**DOI:** 10.3390/healthcare10050867

**Published:** 2022-05-07

**Authors:** Ghadah Asaad Assiri, Bashayr Mohammed Alanazi, Yazed AlRuthia

**Affiliations:** Department of Clinical Pharmacy, College of Pharmacy, King Saud University, P.O. Box 2454, Riyadh 11451, Saudi Arabia; ph.bashayera@gmail.com (B.M.A.); yazeed@ksu.edu.sa (Y.A.)

**Keywords:** prescribing safety indicators, adults, primary care clinic, primary healthcare, electronic health records, non-steroidal anti-inflammatory drugs, prevalence, risk factors

## Abstract

The quality and safety of prescribed drugs can be assessed using prescribing safety indicators (PSIs). This study aimed to estimate the prevalence of PSIs of oral non-steroidal anti-inflammatory drugs (NSAIDs) at primary care clinics of a tertiary care hospital in Saudi Arabia and to identify the risk factors associated with positive PSIs for patients. In this retrospective chart review study, data from the medical records of 450 patients aged ≥18 years, who were prescribed oral NSAIDs, were reviewed and collected manually. Seven PSIs were chosen and defined as follows: prescription of an oral NSAID to any patient with a peptic ulcer; aged ≥75 years; aged ≥65 years with a glomerular filtration rate <60; heart failure; co-prescribed warfarin; co-prescribed aspirin or clopidogrel; or co-prescribed angiotensin-converting enzyme inhibitor/angiotensin II receptor blocker and a diuretic. Patients with positive indicators are at risk of harm from high-risk prescribing. The overall period prevalence of PSIs is 153/450 (34%; 95% CI 29.60–38.39). The overall proportion of PSIs is 202/431 (46.9%; 95% CI 42.12–51.61). The most common safety indicators were for NSAIDs prescribed to patients with heart failure and patients aged ≥65 years with a glomerular filtration rate <60. The elderly and patients using polypharmacy are at increased risk of having at least one positive PSI (OR 5.22; 95% CI 3.32–8.21, *p*-value < 0.001 and OR 2.97; 95% CI 1.17–7.55, *p*-value 0.022, respectively). Patients at risk of harm from high-risk NSAID prescriptions are common in primary care. The elderly and patients on polypharmacy are at increased risk of having at least one positive PSI. Therefore, when NSAIDs are prescribed, it is recommended to weigh the benefits versus the risks for high-risk patients, such as the elderly and those with multiple-drug therapy.

## 1. Introduction

Adverse events caused by medications are a major concern in all healthcare institutions and settings. The majority of hospital admissions are due to drug-related morbidity, which is generally the result of ineffective prescribing and monitoring during the medication management process [1].

Some tools and methods can be used in the medication management process to analyse prescriptions, such as prescribing safety indicators (PSIs), which can assess the safety of medications in the prescribing stage; prescribing analysis and cost (PACT) data, which includes information on prescribing costs, the number of prescribed items, and generic prescribing; and the medication appropriateness index (MAI), which measures the appropriateness of prescribing for each medication for elderly patients [2,3]. The most appropriate tool for risk-of-harm estimations is the PSI, which can be used for the rapid assessment of prescribing safety [2]. Numerous previous studies have used PACT to assess prescribing trends [4,5], and most MAI studies have been conducted on reliability [6,7]. PACT and MAI might not be helpful in risk-of-harm estimations relating to prescription drugs in primary care settings since MAI is time consuming and requires detailed clinical record assessment [6], and PACT is rarely linked to clinical data and mainly focuses on the issue of cost [2,3].

PSIs were defined by Spencer et al. as “statements describing prescribing events that put the patient at risk of harm” [3]. These indicators are tools that assess the quality and safety of prescribed drugs in primary care settings, and a means to measure the risk of harm among vulnerable patient populations who are at elevated risk of harm from high-risk prescribed drugs [8]. Avery et al. and Dreischulte et al. generated and validated different PSIs using the consensus method to ensure the proper use of medications in primary care settings [9,10]. Examples of medication studies regarding PSIs are studies on antiplatelet drugs, beta blockers, opioids, non-steroidal anti-inflammatory drugs (NSAIDs) co-prescribed with warfarin, methotrexate, and antipsychotics [8,9,10].

In some cases, high-risk prescribing indicators are detected, but these indicators are not necessarily inappropriate [2]. For example, healthcare providers may decide to prescribe a medication with a poor safety profile for patients with compromised renal function after discussing the benefits and risks associated with that particular drug with their patients [2]. Whether PSIs are appropriate or not and the occurrence of harm are beyond the scope of this study. A cross-sectional population database analysis of general practices in Scotland found that 13.9% of patients had at least one PSI [8]. Another UK study of general practices using routine electronic medical record data found that the percentage of patients with any PSI fell from 8.5% in 2004 to 5.2% in 2009, which was due to a reduction in high-risk NSAID use [2]. However, the previous rates were related to PSIs of different drug groups.

In this study, the focus is on indicators related to oral NSAIDs, only because the utilisation of NSAIDs is increasing through both self-use (over-the-counter (OTC) medication) and prescriptions from primary care clinics [11,12]. Consequently, we expect a greater risk of harm from NSAIDs than from other groups of medications. Multiple studies have explored prescribing patterns and medication errors in the hospital setting compared to primary healthcare in Saudi Arabia [13,14], and currently, there is much interest in studying prescribing patterns and behaviours [15,16]. Still, more work needs to be conducted on safe prescribing [16,17,18]. Only few studies have explored PSIs in primary care settings [19].

Therefore, exploring and examining PSIs related to NSAIDs, which are a highly prescribed group of analgesics both locally and globally, will lay the groundwork for future studies aimed at developing interventions to prevent NSAID-related PSIs. Thus, the aim of this study is to estimate the prevalence of PSIs related to oral NSAIDs at the primary care clinics of a university-affiliated tertiary care centre in Saudi Arabia and to identify the risk factors of patients with positive PSIs related to oral NSAIDs, which is something that has not been examined before in Saudi healthcare settings.

## 2. Materials and Methods

### 2.1. Study Design and Setting 

#### Reporting

This study followed the Strengthening the Reporting of Observational Studies in Epidemiology (STROBE) checklist and the Reporting of Studies Conducted Using Observational Routinely Collected Health Data (RECORD) statement (see Supplementary S1) [20,21].

A retrospective chart review study was carried out at the primary care clinics of a 1200-bed tertiary care hospital in the central region of Saudi Arabia. The hospital is a multi-disciplinary facility providing primary, secondary, and tertiary care, treating more than 1 million outpatients each year. In addition, approximately 45,966 patients are admitted and approximately 14,231 procedures are performed every year. The primary care clinics provide clinical management, prevention, surveillance and detection, and the maintenance of essential services.

### 2.2. Ethical Considerations

Ethical approval was obtained from the Institutional Review Board (IRB) for Health Sciences Colleges Research on Human Subjects, King Saud University, Riyadh, Saudi Arabia (IRB Approval of Research Project No. E-20-4917). The ethics committee waived the requirement of written informed consent for participation. All data and patient identifiers were fully anonymised (Supplementary S2). To ensure data anonymity and patient confidentiality, patient identifiers were not used in the research; every patient involved in this study received codes and serial numbers known only to the researchers. Permission to use the PSIs was obtained (Supplementary S3).

### 2.3. Participants

**Figure 1:**
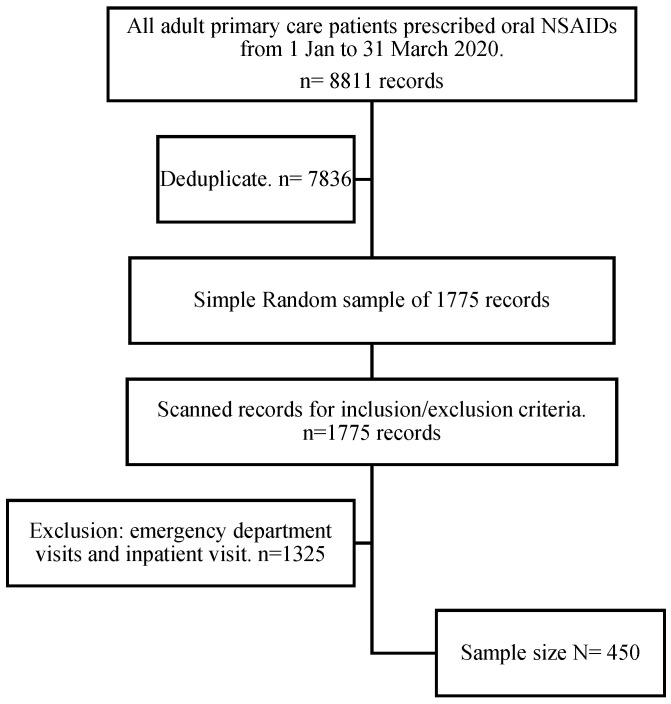
Retrospective chart review study flowchart. NSAIDs, non-steroidal anti-inflammatory drugs.

A pilot study was carried out with 10% of the total sample to ensure data collection and extraction feasibility. A list of all patients on oral NSAIDs who visited a primary care clinic from 1 January 2020 to 31 March 2020 (3-month period of the study) was generated from the electronic health record (EHR) system of the clinics, giving a list of 7836 deduplicated records. Each record was given a code number, and 1775 records were randomly selected using a random number table that was generated using the “simple random sample without replacement” function in STATA (version 14) statistical software. Screening for the eligibility and inclusion criteria was applied to 1775 records by reviewing each patient’s demographic information, medication list, and pre-existing diseases until a sample size of 450 patient records was reached. Finally, a serial number was assigned to each record (see retrospective chart review study flowchart, Figure 1).

The Retrospective chart review was carried out over the 12 weeks following the date of NSAID prescription. The 12-week duration was specified in the PSI tool [2,8].

#### 2.3.1. Baseline Characteristics 

The baseline characteristics taken into account were age, gender, nationality (Saudi, non-Saudi), pre-existing diseases, pre-existing co-prescriptions (e.g., angiotensin-converting enzyme (ACE) inhibitor, angiotensin II receptor blocker (ARB), antiplatelet agent, diuretic, or oral anticoagulant (OAC)), polypharmacy (≥5 medications at any point during the 12-week period) [22], and duration of NSAID use. 

#### 2.3.2. Inclusion Criteria 

Patients considered for inclusion were age 18 years or older, had active medical records, had been seen by a primary care provider at least 3 months prior to the start of data collection, were taking at least one oral NSAID, and had any co-morbid condition (e.g., chronic kidney disease (CKD), diabetes mellitus, heart failure, hypertension, or osteoarthritis).

#### 2.3.3. Exclusion Criteria 

Patients were excluded from the study if they were administering subcutaneous, intramuscular, or injectable NSAIDs, or if they took NSAIDs in an inpatient or emergency healthcare setting.

### 2.4. Variables

a. Outcome variables:

The PSIs measure the risk of drugs prescribed to vulnerable patients, a situation that involves the risk of harm. These drugs can put patients at risk of harm through possible gastrointestinal (GI) bleeding, renal toxicity, or worsening of a heart condition and potential failure because of the patient’s age (≥65 years), pre-existing condition (peptic ulcer, stage 3–5 CKD, or heart failure), or co-prescribed drugs (antiplatelets, OACs or ACE/ARBs, and diuretics, the “triple whammy”) (see Supplementary S4).

Medications under antiplatelet, OAC or ACE inhibitor/ARB, and diuretic groups were medications available in the hospital formulary. In addition, gastroprotective drugs were those in the proton pump inhibitor and H-2 receptor antagonist groups.

The PSIs were developed and validated based on Guthrie’s criteria for high-risk prescribing in general practice through a two-round consensus method using the Delphi process [8,9,10]. In this study, we focused on the seven indicators related to oral NSAIDs: numbers 1, 2, 3, 4, and 7 from Guthrie et al. [2] and numbers 5 and 6 from Guthrie et al. [8]. Each indicator consists of a numerator (patients counted as having high-risk NSAID prescriptions) and a denominator (at-risk patients). Patients with positive indicators are considered to be at risk of harm from high-risk prescribing.

b. Risk factors

The considered risk factors were age, gender, nationality, polypharmacy, and duration of NSAID use. Age, gender, and polypharmacy were evaluated as risk factors by Guthrie et al. and Stocks et al. [8,23]. Studies in Saudi Arabia on high-risk prescribing suggested nationality (Saudi or non-Saudi) as a risk factor [24,25]. Furthermore, we wanted to examine if the duration of NSAID use would trigger a prescribing indicator. This factor was chosen based on previous epidemiological studies [26].

c. Exposure

In this study, we considered exposure to one of the oral NSAIDs available in the hospital formulary: celecoxib, diclofenac, ibuprofen, indomethacin, and meloxicam. 

### 2.5. Data Collection Data Source 

The data collection and extraction were undertaken manually on paper data collection sheets (Supplementary S5) at the primary care clinics in Riyadh, Saudi Arabia, by one of the authors (B.M.A.) as part of her master’s degree project. B.M.A. had access to and training on the EHR system for data collection and extraction. Data collection was carried out from November 2020 to March 2021.

The paper data collection sheet was designed by the research team to include the relevant information related to patient demographics, past medical history, co-prescribed drugs, and NSAID indicators. Data were then transferred to an electronic Excel datasheet and coded.

### 2.6. Bias

A simple random model was employed for sampling in order to avoid selection bias. B.M.A. conducted the data collection. To ensure the reliability of the information, 20% of the data collection was double-checked by a second author (G.A.A.) [27,28]. In addition, to ensure accuracy, all data transferred from the paper forms to the Excel datasheet were double-checked by G.A.A. 

### 2.7. Sample Size Estimation

(1) Retrospective chart review study sample size:

The sample size was calculated based on the assumption that the prevalence of NSAID PSIs is 50%, because no similar studies have previously been conducted in Saudi Arabia [29]. Therefore, the current study sample size was computed using Cochran’s sample size formula. A sample size of 380 records was required to achieve appropriate statistical power (95% confidence interval and 5% margin of error) [29].

(2) Pilot sample size (10% of retrospective chart review total sample size) [30]: 10 × 380/100 = 38 records.
(3) Final adjusted sample size:

The addition of 10–20% records was required to allow adjustment of other factors such as patients excluded for not meeting the inclusion criteria [31]. The final adjusted sample size allowed an excluded files rate of 20% (N1):

N1 = N/(1 − 0.2) = 380/0.8 = 475 records to screen for eligibility.

In this study, we screened 1775 records for eligibility because multiple records met the exclusion criteria and were eventually excluded.

Final sample size: 450 records.

### 2.8. Statistical Methods

To illustrate the respondents’ demographic characteristics, descriptive statistics were calculated using frequencies and percentages. The overall period prevalence rate was calculated as the number of patients experiencing one or more PSIs at any time during the 12-week period/total number of patients in study population ×100. The proportion of each PSI was calculated as the number of positive numerators for each PSI during the 12-week period/number of positive denominators for each PSI × 100.

The overall PSI proportion was calculated as the total number of positive numerators for all PSIs during the12-week period/ total number of positive denominators for all PSIs × 100. For continuous variables, the results are presented as mean ± standard deviation. For categorical variables, the results are presented as counts with percentages.

To evaluate the association of risk factors and outcome, logistic regression analysis was conducted. The dependent variable was the presence/absence of the outcome (PSI). The patient- and medication-related independent variables were age in years, gender, nationality, polypharmacy (≥5 medications), and duration of NSAID use. All independent variables were binary (0 = no; 1 = yes), except for the duration of NSAID use, which had 5 categories (1–30, 31–90, 91–180, 181–270, and ≥271 days). Significance at *p* < 0.05 and 95% CI were used. The analysis was conducted using STATA (version 14, StataCorp LLC, College Station, TX, USA) statistical software.

### 2.9. Data Access and Cleaning Methods

The Excel data sheet was checked for outliers or errors in data entry. For the PSI outcomes, several data checks were used to ensure the following: the ages of included patients met the inclusion criteria for the relevant PSI outcome; the co-prescribed drugs met the criteria for the relevant outcome measure; the co-existing conditions met the criteria for the relevant outcome measure; cases labelled as numerators met the criteria for being numerators; and cases labelled as denominators met the criteria for being denominators.

### 2.10. Patient and Public Involvement

Patients and the public were not involved in the designing, carrying out, reporting, or disseminating of the plans of this research.

## 3. Results

A total of 450 records met the inclusion criteria and were included in the retrospective chart review study (see Figure 1).

### 3.1. Demographic Characteristics

The demographic characteristics show that 64% of patients were between 18 and 64 years old, with a mean age of 62.01 years (standard deviation (SD) 11.15), and that 70% were female. All patients were Saudi. Most of the patients (90%) were using five or more prescription medications. Celecoxib 200 mg was the most used NSAID in this study population (53%). None of the patients were on indomethacin. More than 60% of patients had one of the following conditions: diabetes mellitus, dyslipidaemia, or hypertension (Table 1).

NSAIDs were prescribed for arthritic conditions and different types of pain in the shoulders, back, joints, and muscles, as well as migraine headaches and fibromyalgia.

### 3.2. Prescribing Safety Indicator Rate

The overall period prevalence rate was (153/450) × 100= 34% (95% CI 29.60–38.39). The PSIs with the highest proportions were number 6, NSAID prescribed for patients over 65 with an estimated glomerular filtration rate <60, and number 7, NSAID prescribed for patients with heart failure, both at 100%. The overall proportion of PSIs was (202/431) × 100 = 46.9% (95% CI 42.12–51.61) (Table 2).

### 3.3. Risk Factors

Patients ≥ 65 years of age were estimated to be five times more likely to have at least one positive PSI than those aged 18–64 years (OR 5.22; 95% CI 3.32–8.21, *p*-value < 0.001).

Patients using polypharmacy were estimated to be approximately three times more likely to have at least one positive PSI than those not using polypharmacy (OR 2.97; 95% CI 1.17–7.55, *p*-value 0.022). 

In contrast, female patients and patients prescribed NSAIDs for 1–3 months or 3–6 months were estimated to be 51, 56, and 64% less likely to have at least one positive PSI, which was statistically significant (Table 3).

## 4. Discussion

This study aimed to estimate the prevalence of PSIs associated with oral NSAID prescriptions using EHRs at primary care clinics of a university-affiliated tertiary care centre in Saudi Arabia and to identify the risk factors associated among patients with positive PSIs. PSIs are common at primary care clinics, posing a significant threat to patient safety and making them a prime target for improvement. The overall period prevalence of PSIs is 34% (95% CI 29.60–38.39). The overall proportion of PSIs is 46.9% (95% CI 42.12–51.61). This is higher than the proportion identified in a UK study, which found that their composite indicator, defined as “prescription of an NSAID to a person with any peptic ulcer, aged 75 years or over, heart failure, co-prescribed warfarin or co-prescribed aspirin or clopidogrel”, varied between general practices ranging from 4.1 to 21% [2]. The difference between the two studies could be due to the different methods of data extraction. The most common indicators in our study are NSAIDs prescribed to patients with heart failure and patients aged ≥65 years with an estimated glomerular filtration rate <60. Patients ≥65 years of age and using polypharmacy were statistically significantly more likely to have at least one positive PSI. This is in agreement with a previous study that examined the prevalence of PSIs in general practice setting in the UK using the Clinical Practice Research Datalink of more than 940,000 patients [23]. Female patients and patients prescribed NSAIDs for 1–3 months or 3–6 months were statistically significantly less likely to have at least one positive PSI.

Comparing our results with those of PSIs studies conducted on general practices in the UK, the proportion of patients identified at risk for each PSI was higher in our study for PSI 2, NSAID prescribed to a person aged ≥75 years [2,23]; PSI 3, NSAID prescribed with antiplatelets [2]; PSI 4, NSAID with OAC [2,8,23]; PSI 5, NSAID prescribed to a person aged ≥65 years with ACE inhibitor/ARB and a diuretic [8]; PSI 6, NSAID prescribed to a person aged ≥65 with GFR <60 [8]; and PSI 7, NSAID prescribed to a person with heart failure [8,23]. However, the proportion of patients identified at risk for each PSI was lower in our study for PSI 1, NSAID with a peptic ulcer [2,8,23]. The difference in the proportion of indicators between our study and the UK studies could be related to differences in prescribing patterns and the methods of data collection; hence, the level of risk associated with PSIs in primary care may vary [23].

A previous study investigated an area similar to that of the current research: a retrospective cohort study was conducted in Saudi Arabia in 2019 to investigate clinically important prescription and monitoring errors among adults managed in family medicine clinics [24]. However, it is not possible to compare the overall prevalence between the two studies because of the differences between outcomes. Three of their outcome measures had the same numerator and denominator, and we were able to compare those results as follows: PSI 1, NSAID with a peptic ulcer: none in either study; PSI 4, NSAID with OAC: we found a higher proportion, 28.6 vs. 6.3%; and PSI 7, NSAID with heart failure: we also found a higher proportion, 100 vs. 21.4% [24]. The higher proportions in our study compared to Assiri et al.’s study could be due to variations between the samples of the two studies. Their study sample involved patients who were receiving at least one prescribed or OTC medication, whereas our study sample involved patients who were prescribed at least one NSAID, which could have triggered more PSIs.

This study has several identifiable strengths. The list of PSIs used in this study was generated and validated using the consensus method to ensure the proper use of medications in primary care [9,10]. Random sampling was employed to decrease selection bias. Additionally, this is the first retrospective chart review study to focus on a pre-specified list of PSIs for NSAIDs involving patients in primary care in Saudi Arabia.

The limitations of this study are as follows: first, the results of this study cannot be generalised to the primary care setting in Saudi Arabia since it is a single-centre study. Second, there is a risk of information bias, as the investigators relied on only EHR information for the identification and assessment of the PSIs. Third, we were not able to see if being Saudi or not was associated with having any PSIs, because none of our patients were non-Saudis. Fourth, the data collection in this study was restricted to a 3-month period, which was suitable for the timetable of the master project and in line with the COVID-19 pandemic, so many factors could have affected our results, such as seasonal variations in prescribing and variations between clinical settings in the region. These two factors were not evaluated in this study; further studies are required to comprehensively assess such factors and PSIs in primary care. 

More research could be conducted to replicate this study in different regions of Saudi Arabia in order to enable generalisation of the results. More research is also needed to understand the patient perspective on the risks and benefits of high-risk NSAID prescribing. Educational workshops for pharmacy students and primary care and community pharmacists are needed to ensure an understanding of indicators showing that patients may be at risk of harm. In addition, optimising those indicators for the electronic record system to alert physicians in case of positive indicators and prevent exposing patients to the risk of harm should be made a priority [32,33].

In the case of patients buying NSAIDs as self-medication from a community pharmacy or physicians prescribing them in primary care, pharmacists and/or prescribing physicians must ask about any pre-existing conditions or co-prescribed drugs that could increase the risk of harm. Once apprised of that information, they should provide advice regarding the benefits versus risks and counsel patients in order to ensure the safe and proper use of medications. In addition, the active role of patients taking responsibility to ensure the safety of their self-care treatment should not be ignored. This goal could be met by facilitating the implementation of online portals that allow patients to access their own medical records in order to enhance their communication with healthcare providers [34,35].

## 5. Conclusions

The use of oral NSAIDs for high-risk prescribing in primary care is common. The overall period prevalence of PSIs related to oral NSAIDs is higher than expected, particularly among elderly patients and those with polypharmacy. It is highly recommended to weigh the benefits and risks of using oral NSAIDs in order to ensure the safe and proper use of these medications. 

## Figures and Tables

**Table 1:** Demographic characteristics.

Outcome	Categories	Number (%)
Age	18–64 years	288 (64)
	≥65 years	162 (36)
	Mean age: 62; standard deviation 11.2	-
Gender	Male	132 (29.3)
	Female	318 (70.7)
Nationality	Saudi	450 (100)
	Non-Saudi	0 (0)
Polypharmacy	Yes, ≥5 concurrent medications	407 (90.4)
	No, 1–4 concurrent medications	43 (9.6)
NSAIDs	Celecoxib 200 mg	238 (52.9)
	Diclofenac 50 mg	36 (8)
	Ibuprofen 400 mg	18 (4)
	Meloxicam 7.5 mg	40 (8.9)
	Meloxicam 15 mg	118 (26.2)
Duration of NSAID use	1–30 days	114 (25.3)
	31–90 days	277(61.6)
	91–180 days	54 (12)
	181–270 days	3 (0.7)
	≥270 days	2 (0.4)
Pre-existing conditions		
Arthritic disorder	Osteoarthritis	191 (42.4)
Cardiac and vascular disorders	Dyslipidaemia	276 (61.3)
	Essential hypertension	338 (75.1)
	Heart failure	7 (1.6)
Endocrine disorder	Diabetes mellitus	316 (70.2)
Gastrointestinal disorder	Ulcer	3 (0.7)
Renal disorder	Chronic kidney disease	50 (11.1)
Co-prescribed drugs		
	Oral anticoagulant	14 (3.1)
	Antiplatelet	200 (44.4)
	Aspirin	185 (92.5)
	Clopidogrel	15 (7.5)
	ACE/ARB	230 (51.1)
	Diuretics	101 (22.4)

ACE, angiotensin-converting enzyme; ARB, angiotensin II receptor blocker.

**Table 2:** Proportion of prescribing safety indicators and period prevalence at patient level (described using numerator, denominator, and percentage). (Adopted from Guthrie et al. [2,8].)

	Prescribing Safety Indicator (PSI) Name	Numerator Definition	Number	Denominator Definition	Number	Proportion of PSI (%); 95%CI
1	Non-steroidal anti-inflammatory drug (NSAID) prescribed to person with history of peptic ulcer, without co-prescription of gastroprotection	Prescribed oral NSAID during quarter and not prescribed gastroprotective drug in 12 weeks before NSAID prescription	0	Diagnosed with peptic ulcer before the quarter	3	0
2	NSAID prescribed to person aged 75 years or over, without co-prescription of gastroprotection	Prescribed oral NSAID during quarter and not prescribed a gastroprotective drug in 12 weeks before NSAID prescription	20	Age 75 years before the quarter	62	32.3; 20.29–44.23
3	NSAID prescribed to person taking an antiplatelet drug, without co-prescription of gastroprotection	Prescribed oral NSAID during quarter and not prescribed gastroprotective drug in 12 weeks before NSAID prescription	92	Prescribed antiplatelet drug during quarter	200	46; 39.03–52.97
4	NSAID prescribed to person taking an oral anticoagulant (OAC), without co-prescription of gastroprotection	Prescribed oral NSAID during quarter and not prescribed gastroprotective drug in 12 weeks before NSAID prescription	4	Prescribed OAC during quarter	14	28.6; 1.50–55.64
5	NSAID prescribed to person aged 65 years or over prescribed angiotensin-converting enzyme (ACE) inhibitor/angiotensin II receptor blocker (ARB) and diuretic (“‘triple whammy”)	Prescribed oral NSAID in same quarter	34	Age 65 years or over before start of quarter and prescribed ACE inhibitor/ARB and diuretic during quarter	100	34; 24.55–43.45
6	NSAID prescribed to patient aged over 65 years with estimated glomerular filtration rate (GFR) <60	Prescribed NSAID during quarter	45	No. of patients aged ≥65 years with stage 3, 4, or 5 renal impairment (estimated GFR <60)	45	100; 100–100
7	NSAID prescribed to patient with heart failure	Prescribed NSAID during quarter	7	Diagnosed with heart failure at time of last prescription	7	100; 100–100
	Overall proportion of prescribing safety indicator	Total number of positive numerators	202	Total number of positive denominators	431	46.9; 42.12–51.61
	Overall period prevalence	Number of patients with at least one positive PSI	153	Total number of patients (N)	450	34; 29.60–38.39

ACE, angiotensin-converting enzyme; ARB, angiotensin II receptor blocker; GFR, glomerular filtration rate; NSAID, non-steroidal anti-inflammatory drug; OAC, oral anticoagulant; PSI, prescribing safety indicator. Quarter: 3-month period of study (January to March 2020). Renal impairment stages: stage 1, with normal or high glomerular filtration rate (GFR > 90 mL/min); stage 2, mild CKD (GFR = 60–89 mL/min); stage 3A, moderate CKD (GFR = 45–59 mL/min); stage 3B, moderate CKD (GFR = 30–44 mL/min); stage 4, severe CKD (GFR = 15–29 mL/min); stage 5, end-stage CKD (GFR <15 mL/min).

**Table 3:** Association between risk factors and patients with at least one positive prescribing safety indicator (data obtained from logistic regression model).

Risk Factor		OR; 95% CI	*p*-Value
Age (≥65 or 18–64 years)		5.22; 3.32–8.21	<0.001 *
Gender (female or male)		0.49; 0.31–0.79	0.003 *
Polypharmacy (yes or no)		2.97; 1.17–7.55	0.022 *
Duration of NSAID use	31–90 days	0.44; 0.26–0.74	0.002 *
	91–180 days	0.36; 0.16–0.82	0.015 *
	181–270 days	1	
	≥270 days	0.32; 0.02–5.56	0.431
	Overall	0.29; 0.11–0.79	0.017 *

NA, no association. OR = 1. * *p*-value significant at < 0.05.

## Data Availability

All available data, including raw data and codes, can be obtained by contacting the corresponding author.

## Supplementary Materials

The following supporting information can be downloaded at https://www.mdpi.com/article/10.3390/healthcare10050867/s1: Supplementary S1. RECORD statement: checklist of items, extended from STROBE statement, that should be reported in observational studies using routinely collected health data. Supplementary S2. Institutional review board research approval. Supplementary S3. Permission to use prescribing safety indicators. Supplementary S4. Prescribing safety indicators for NSAIDs. Supplementary S5. Data collection sheet.
