# Supplementary material for: The Prevalence of High-Risk Prescribing of Oral Non-Steroidal Anti-Inflammatory Drugs in Primary Healthcare: A Single-Centre Retrospective Chart Review Study
Source: Healthcare (Basel). 2022 May 7;10(5):867. doi: 10.3390/healthcare10050867 (PMC9142064; doi:10.3390/healthcare10050867)
Supplement: Supplementary file 1 [file healthcare-10-00867-s001.zip › healthcare-1633111-supplementary.pdf]

Supplementary S1: The RECORD statement – checklist of items, extended from the STROBE statement, that should be reported in observational studies using routinely collected health data*.

	Item No.	STROBE items	Location in manuscript where items are reported	RECORD items	Location in manuscript where items are reported
**Title and abstract**					
	1	(a) Indicate the study's design with a commonly used term in the title or the abstract (b) Provide in the abstract an informative and balanced summary of what was done and what was found	1	RECORD 1.1: The type of data used should be specified in the title or abstract. When possible, the name of the databases used should be included.<br><br>RECORD 1.2: If applicable, the geographic region and timeframe within which the study took place should be reported in the title or abstract.<br><br>RECORD 1.3: If linkage between databases was conducted for the study, this should be clearly stated in the title or abstract.	1

**Introduction**					
Background rationale	2	Explain the scientific background and rationale for the investigation being reported	1,2		
Objectives	3	State specific objectives, including any prespecified hypotheses	2		
**Methods**					
Study Design	4	Present key elements of study design early in the paper	2		
Setting	5	Describe the setting, locations, and relevant dates, including periods of recruitment, exposure, follow-up, and data collection	2		
Participants	6	*(a) Cohort study* - Give the eligibility criteria, and the sources and methods of selection of participants. Describe methods of follow-up.<br><br>*Case-control study* - Give the eligibility criteria, and the sources and methods of case ascertainment and control selection. Give the	3	RECORD 6.1: The methods of study population selection (such as codes or algorithms used to identify subjects) should be listed in detail. If this is not possible, an explanation should be provided.	3

		rationale for the choice of cases and controls.<br><br>*Cross-sectional study* - Give the eligibility criteria, and the sources and methods of selection of participants.<br><br>*(b) Cohort study* - For matched studies, give matching criteria and number of exposed and unexposed<br><br>*Case-control study* - For matched studies, give matching criteria and the number of controls per case		RECORD 6.2: Any validation studies of the codes or algorithms used to select the population should be referenced. If validation was conducted for this study and not published elsewhere, detailed methods and results should be provided.<br><br>RECORD 6.3: If the study involved linkage of databases, consider use of a flow diagram or other graphical display to demonstrate the data linkage process, including the number of individuals with linked data at each stage.	
Variables	7	Clearly define all outcomes, exposures, predictors, potential confounders, and effect modifiers. Give diagnostic criteria, if applicable.	4	RECORD 7.1: A complete list of codes and algorithms used to classify exposures, outcomes, confounders, and effect modifiers should be provided. If these cannot be reported, an explanation should be provided.	4
Data sources/ measurement	8	For each variable of interest, give sources of data and details of methods of assessment (measurement).	4		

		Describe comparability of assessment methods if there is more than one group			
Bias	9	Describe any efforts to address potential sources of bias	5		
Study size	10	Explain how the study size was arrived at	5		
Quantitative variables	11	Explain how quantitative variables were handled in the analyses. If applicable, describe which groupings were chosen, and why	5		
Statistical methods	12	(a) Describe all statistical methods, including those used to control for confounding<br><br>(b) Describe any methods used to examine subgroups and interactions<br><br>(c) Explain how missing data were addressed<br><br>(d) *Cohort study* - If applicable, explain how loss to follow-up was addressed	5		

		Case-control study - If applicable, explain how matching of cases and controls was addressed.<br><br>*Cross-sectional study* - If applicable, describe analytical methods taking account of sampling strategy<br><br>(e) Describe any sensitivity analyses			
Data access and cleaning methods		..		RECORD 12.1: Authors should describe the extent to which the investigators had access to the database population used to create the study population.<br><br>RECORD 12.2: Authors should provide information on the data cleaning methods used in the study.	5
Linkage		..		RECORD 12.3: State whether the study included person-level, institutional-level, or other data linkage across two or more databases. The methods of linkage and methods of linkage quality evaluation should be provided.	-

Results					
Participants	13	(a) Report the numbers of individuals at each stage of the study (*e.g.*, numbers potentially eligible, examined for eligibility, confirmed eligible, included in the study, completing follow-up, and analysed)<br><br>(b) Give reasons for non-participation at each stage.<br><br>(c) Consider use of a flow diagram	6	RECORD 13.1: Describe in detail the selection of the persons included in the study (*i.e.,* study population selection) including filtering based on data quality, data availability and linkage. The selection of included persons can be described in the text and/or by means of the study flow diagram.	6
Descriptive data	14	(a) Give characteristics of study participants (*e.g.*, demographic, clinical, social) and information on exposures and potential confounders<br><br>(b) Indicate the number of participants with missing data for each variable of interest<br><br>(c) *Cohort study* - summarise follow-up time (*e.g.*, average and total amount)	6		

Outcome data	15	*Cohort study* - Report numbers of outcome events or summary measures over time<br><br>*Case-control study* - Report numbers in each exposure category, or summary measures of exposure<br><br>*Cross-sectional study* - Report numbers of outcome events or summary measures	7		
Main results	16	(a) Give unadjusted estimates and, if applicable, confounder-adjusted estimates and their precision (e.g., 95% confidence interval). Make clear which confounders were adjusted for and why they were included.<br><br>(b) Report category boundaries when continuous variables were categorized<br><br>(c) If relevant, consider translating estimates of relative risk into absolute risk for a meaningful time period	7		
Other analyses	17	Report other analyses done—e.g., analyses of subgroups and	8		

		interactions, and sensitivity analyses			
**Discussion**					
Key results	18	Summarise key results with reference to study objectives	8		
Limitations	19	Discuss limitations of the study, taking into account sources of potential bias or imprecision. Discuss both direction and magnitude of any potential bias	8,9	RECORD 19.1: Discuss the implications of using data that were not created or collected to answer the specific research question(s). Include discussion of misclassification bias, unmeasured confounding, missing data, and changing eligibility over time, as they pertain to the study being reported.	9
Interpretation	20	Give a cautious overall interpretation of results considering objectives, limitations, multiplicity of analyses, results from similar studies, and other relevant evidence	9		
Generalisability	21	Discuss the generalisability (external validity) of the study results	9		
**Other Information**					

Funding	22	Give the source of funding and the role of the funders for the present study and, if applicable, for the original study on which the present article is based	10		
Accessibility of protocol, raw data, and programming code		..		RECORD 22.1: Authors should provide information on how to access any supplemental information such as the study protocol, raw data, or programming code.	10

*(1)(2)

Supplementary S2: IRB research approval

Kingdom of Saudi Arabia
King Saudi University (034)
P.O. Box 7805 Riyadh 11472
Tel: +966 11 467 00 11
Fax: +96611 467 19 92
http://medicalcity.ksu.edu.sa

المملكة العربية السعودية
جامعة الملك سعود (٠٣٤)
ص.ب ٧٨٠٥ الرياض ١١٤٧٢
هاتف: +٩٦٦١١٤٦٧٠٠١١
فاكس: +٩٦٦١١٤٦٧١٩٩٢

جامعة الملك سعود
King Saud University
المدينة الطبية الجامعية

26.07.2020 (05.12.1441)
Ref. No. 20/0399/IRB

To: **Dr. Ghadah Assiri**
Assistant Professor
Department of Clinical Pharmacy
College of Pharmacy, King Saud University
Email: gassiri@ksu.edu.sa
Principal Investigator

Cc: Bashayr Mohamed Alanazi -
E-mail: ph.bashayera@gmail.com / 441203941@student.ksu.edu.sa
Co-investigator

Subject: **Approval of Amendment of Research Project No. E-20-4917**

Study Title: "The Prevalence of Prescribing Safety Indicators of Oral Nonsteroidal Anti-inflammatory Drugs in Primary Health Care: A Single-Center Retrospective Cohort Study."

Review Category: Expedite

Dear Dr. Ghadah Assiri,

Reference to your request for the approval of the amendment done on the above-mentioned research project which was initially reviewed and approved on 28 June 2020 (07 Dhu Al- Qa'dah 1441), please be informed that the IRB has no objection toward your request to change the study data collection date from January to March 2020 instead of April 2020 to June 2020 as stated in the approved proposal as patients might not be coming to the clinic during this time due to the COVID-19 pandemic.

You may continue with the conduct of this study with the above-listed additional study personnel. Please be informed that in conducting this study, you as the principal investigator, are required to abide by the rules and regulations of the Government of Saudi Arabia, the KSUMC IRB policies and procedures and the ICH-GCP Guidelines. The IRB mandates regular submission of study progress report every six months by the primary investigator. Otherwise, project approval will be suspended.

We wish you success in your research.

Thank you!

Sincerely yours,

Prof. Abdulrahman AlSultan
Chairman of IRB
Health Sciences Colleges Research on Human Subjects
King Saud University College of Medicine
P. O. B ox 7805 Riyadh 11472 K.S.A.
Email: aalsultan1@ksu.edu.sa

College Medicine Institutional Review Board (IRB)

/beth

Supplementary S3: Permission to use the prescribing safety indicators

Copyright Clearance Center RightsLink®

Home | Help | Live Chat | Ghadah Assiri

SPRINGER NATURE

The rising tide of polypharmacy and drug-drug interactions: population database analysis 1995–2010

Author: Bruce Guthrie et al

Publication: BMC Medicine

Publisher: Springer Nature

Date: Apr 7, 2015

Copyright © 2015, Guthrie et al.; licensee BioMed Central.

Creative Commons

The request you have made is considered to be non-commercial/educational. As the article you have requested has been distributed under a Creative Commons license (Attribution-Noncommercial), you may reuse this material for non-commercial/educational purposes without obtaining additional permission from Springer Nature, providing that the author and the original source of publication are fully acknowledged (please see the article itself for the license version number). You may reuse this material without obtaining permission from Springer Nature, providing that the author and the original source of publication are fully acknowledged, as per the terms of the license. For license terms, please see http://creativecommons.org/

BACK | CLOSE WINDOW

© 2021 Copyright - All Rights Reserved | Copyright Clearance Center, Inc. | Privacy statement | Terms and Conditions
Comments? We would like to hear from you. E-mail us at customercare@copyright.com

Supplementary S4: Prescribing safety indicators for NSAIDs. (Adopted from Guthrie et al. (3,4)**.)**

PSI No.	Prescribing safety indicator (PSI) name	Numerator definition	Denominator definition	Risk of harm
1	Non-steroidal anti-inflammatory drug (NSAID) prescribed to person with history of peptic ulcer, without co-prescription of gastroprotection	Prescribed oral NSAID during quarter and not prescribed gastroprotective drug in 12 weeks before NSAID prescription	Diagnosed with peptic ulcer before quarter	Significant risk of gastrointestinal (GI) bleeding
2	NSAID prescribed to person age 75 years or over, without co-prescription of gastroprotection	Prescribed oral NSAID during quarter and not prescribed gastroprotective drug in 12 weeks before NSAID prescription	Age 75 years before quarter	Significant risk of GI bleeding
3	NSAID prescribed to person taking an antiplatelet drug, without co-prescription of gastroprotection	Prescribed oral NSAID during quarter and not prescribed gastroprotective drug in 12 weeks before NSAID prescription	Prescribed antiplatelet drug during quarter	Significant risk of GI bleeding
4	NSAID prescribed to person taking an oral anticoagulant (OAC), without co-prescription of gastroprotection	Prescribed oral NSAID during quarter and not prescribed gastroprotective drug in 12 weeks before NSAID prescription	Prescribed OAC during quarter	Significant risk of GI bleeding
5	NSAID prescribed to person age 65 years or over taking an angiotensin-converting enzyme (ACE) inhibitor/ angiotensin II receptor blocker	Prescribed oral NSAID in same quarter	Age 65 years or over before start of quarter and prescribed ACE inhibitor/ARB and diuretic during quarter	Renal toxicity

	(ARB) and a diuretic ("triple whammy")			
6	NSAID prescribed to patient over 65 years with estimated glomerular filtration rate (GFR) <60	Prescribed NSAID during quarter	Age ≥65 years with stage 3, 4 or 5 renal impairment (estimated GFR <60)	Renal toxicity
7	NSAID, prescribed to patient with heart failure	Prescribed NSAID during quarter	Diagnosed with heart failure recorded at time of last prescription	Worsening of heart failure

ACE, angiotensin-converting enzyme; ARB, angiotensin II receptor blocker; GFR, glomerular filtration rate; GI, gastrointestinal; NSAID, non-steroidal anti-inflammatory drug; OAC, oral anticoagulant; PSI, prescribing safety indicator. Quarter: 3-month period of study (January to March 2020). Renal impairment stages: stage 1, with normal or high glomerular filtration rate (GFR > 90 mL/min); stage 2, mild CKD (GFR = 60-89 mL/min); stage 3A, moderate CKD (GFR = 45-59 mL/min); stage 3B, moderate CKD (GFR = 30-44 mL/min); stage 4, severe CKD (GFR = 15-29 mL/min); stage 5, end-stage CKD (GFR <15 mL/min).

Supplementary S5: Data collection sheet

A: demographic and basic information

Patient characteristics				
**Patient code**				
**Age**	____ Years			
**Gender**	M		F	
**Nationality**	Saudi		Non-Saudi	
**Diagnosis or past medical history**				
		Hear Failure	Stage	
		Peptic ulcer		
		Osteoarthritis		
		Back pain		
		Chronic kidney disease	Stage	
		Hypertension		
		Type 1 diabetes mellitus		

**If the patient had a history of the following:**	
Peptic ulcer:	(Outcome 1)
Patient aged ≥ 75 years:	(Outcome 2)
eGFR < 60	(Outcome 6)
Heart Failure	(Outcome 7)
**If the patient was on the following medications**	
Antiplatelet	(Outcome 3)
Oral anti-coagulant	(Outcome 4)
Angiotensin-converting enzyme (ACE) inhibitors or diuretics –	(Outcome 5)

			Type 2 diabetes mellitus		
			Other:		
**Polypharmacy at any point** (≥ 5 medications**)**	Yes			No	

Numerator		Yes/no	Denominator	Yes/no	Comment
**The NSAIDs indicators**					
**1**	NSAID prescribed to person with history of peptic ulcer, without co prescription of gastro protection.		Diagnosed with peptic ulcer before the start of the quarter.		
**2**	NSAID prescribed in patient over 75 years without gastro protection.		Aged 75 years and over before the start of the quarter.		
**3**	NSAID prescribed to person prescribed an antiplatelet drug, without co-prescription of gastro protection.		Prescribed an antiplatelet drug during the quarter.		
**4**	Prescribed an oral NSAID during the quarter and not prescribed a gastroprotective drug in the 12 weeks before NSAID prescription.		Prescribed an oral anticoagulant during the quarter.		
**5**	Prescribed an oral NSAID during the quarter.		Aged 65 years and over before the start of the quarter and prescribed an ACE inhibitor/ARB and a diuretic during the quarter. (the 'triple whammy').		
**6**	Prescribed NSAID during the quarter.		No of patients aged ≥65 years with stage 3, 4, or 5 renal impairment* (estimated glomerular filtration rate <60).		

**7**	Prescribed NSAID during the quarter.		Diagnosed with heart failure before the start of the quarter.		

Table: NSAID prescribing safety indicators.**

*Renal impairment stages: stage 1 with normal or high Glomerular Filtration Rate (GFR) (GFR > 90 mL/min). Stage 2 Mild CKD (GFR = 60-89 mL/min). Stage 3A Moderate CKD (GFR = 45-59 mL/min). Stage 3B Moderate CKD (GFR = 30-44 mL/min). Stage 4 Severe CKD (GFR = 15-29 mL/min). Stage 5 End Stage CKD (GFR <15 mL/min).

**(3)(4)

1. Vandenbroucke JP, von Elm E, Altman DG, Gøtzsche PC, Mulrow CD, Pocock SJ, et al. Strengthening the Reporting of Observational Studies in Epidemiology (STROBE): Explanation and elaboration. Int J Surg [Internet]. 2014 Dec [cited 2021 Apr 1];12(12):1500–24. Available from: https://linkinghub.elsevier.com/retrieve/pii/S1743919114002131

2. Benchimol EI, Smeeth L, Guttmann A, Harron K, Moher D, Petersen I, et al. The REporting of studies Conducted using Observational Routinely-collected health Data (RECORD) Statement. PLOS Med [Internet]. 2015 Oct 6 [cited 2021 Apr 1];12(10):e1001885. Available from: https://dx.plos.org/10.1371/journal.pmed.1001885

3. Guthrie B, McCowan C, Davey P, Simpson CR, Dreischulte T, Barnett K. High risk prescribing in primary care patients particularly vulnerable to adverse drug events: cross sectional population database analysis in Scottish general practice. BMJ [Internet]. 2011 Jun 21;342(jun21 1):d3514–d3514. Available from: https://www.bmj.com/lookup/doi/10.1136/bmj.d3514

4. Guthrie B, Yu N, Murphy D, Donnan PT, Dreischulte T. Measuring prevalence, reliability and variation in high-risk prescribing in general practice using multilevel modelling of observational data in a population database. Heal Serv Deliv Res [Internet]. 2015 Oct;3(42):1–140. Available from: https://www.journalslibrary.nihr.ac.uk/hsdr/hsdr03420/
